# Recent research hotspots in sequencing and the pancreatic neuroendocrine tumor microenvironment

**DOI:** 10.20892/j.issn.2095-3941.2023.0284

**Published:** 2023-09-28

**Authors:** Junfeng Xu, Wuhu Zhang, Xin Lou, Zeng Ye, Yi Qin, Jie Chen, Xiaowu Xu, Xianjun Yu, Shunrong Ji

**Affiliations:** 1 Department of Pancreatic Surgery, Fudan University Shanghai Cancer Center, Shanghai 200032, China; 2 Center for Neuroendocrine Tumors, Fudan University Shanghai Cancer Center, Shanghai 200032, China; 3 Department of Oncology, Shanghai Medical College, Fudan University, Shanghai 200032, China; 4 Shanghai Pancreatic Cancer Institute, Shanghai 200032, China; 5 Pancreatic Cancer Institute, Fudan University, Shanghai 200032, China

Multiple endocrine neoplasia 1 (MEN1) syndrome, a disease arising from a genetic predisposition to tumor development caused by MEN1 loss-of-function mutations, is characterized by the combined occurrence of neuroendocrine tumors in multiple human organs. With advances in diagnostic technologies and improvements in living standards, the detection rate of sporadic pancreatic neuroendocrine tumors (PanNETs) has recently increased. Clinical research and treatment approaches for PanNETs have made remarkable progress. However, the heterogeneity and the mechanisms underlying PanNET malignancy have not yet been fully elucidated. Recurrence and metastasis remain major issues faced by clinicians. In-depth research on the biological behavior of PanNETs is necessary to improve treatment efficacy and precision. With molecular biology advances, new basic research findings may have potential clinical translational value for PanNETs^1,2^. Specifically, advances in high-throughput sequencing technologies have greatly facilitated the identification of new biomarkers, and enhanced understanding of the genetic and molecular signatures of PanNETs. Herein, we summarize the characteristics of PanNETs and focus on clinical translational research based on multiomics sequencing in PanNETs. In addition, research hotspots in the tumor microenvironment and using sequencing to explore the pathogenesis of PanNETs are described, to highlight their potential clinical translational value (**Figure 1**).

**Figure 1:**
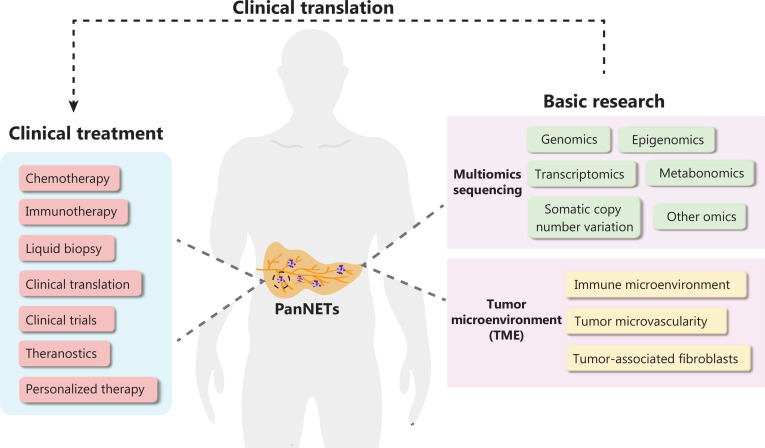
Summary figure describing research hotspots in pancreatic neuroendocrine tumors. Multiomics sequencing research includes genomics, transcriptomics, metabonomics, somatic copy number variation analysis, and other omics. Tumor microenvironment research focuses on the immune microenvironment, tumor microvascularity, and tumor-associated fibroblasts.

## Overview of PanNETs

Pancreatic tumors are classified into two main types—exocrine tumors and neuroendocrine tumors—in the 2017 World Health Organization classification system. More than 90% of pancreatic tumors are exocrine tumors that arise from the exocrine cells of the pancreas and secrete digestive enzymes. Exocrine tumors are further classified according to their cells of origin; the most common form is pancreatic ductal adenocarcinoma, and rare forms include acinar cell carcinoma, intraductal papillary-mucinous neoplasms, and mucinous cystic neoplasms with invasive adenocarcinoma. Less than 10% of pancreatic tumors are neuroendocrine tumors; most are nonfunctional (NF-) PanNETs, and the others are functional PanNETs, including insulinomas, glucagonomas, gastrinomas, somatostatinomas, and VIPomas, classified according to the active hormones that they over-produce.

PanNETs are highly heterogeneous and are histopathologically divided into well-differentiated PanNETs and poorly differentiated pancreatic neuroendocrine carcinomas (PanNECs)^3^. PanNECs are much more aggressive than PanNETs. According to the 8th edition of the American Joint Committee on Cancer staging system for PanNETs and the European Neuroendocrine Tumors Society system, the characteristics, diagnosis, treatment approaches, and prognoses for PanNETs greatly vary by location, stage, grade, differentiation level, and proliferation index. Advances in molecular imaging techniques, such as fluorine 18 (^18^F)-fluorodeoxyglucose PET/CT and somatostatin receptor scintigraphy (indium 111 [^111^In] and gallium 68 [^68^Ga] tetraazacyclododecane tetraacetic acid-octreotate scanning) have led to marked improvements in the assessment and detection of PanNETs. For well-differentiated PanNETs, surgery is the first treatment choice. Peptide receptor radionuclide therapy is a novel treatment modality in which octreotide, a somatostatin analog, is used as a carrier to deliver radionuclides, such as lutetium 177 (^177^Lu) and ^90^Y, to tumor cells expressing somatostatin receptors, thus leading to tumor cell death. This treatment, compared with other therapies, effectively decreases tumor size and increases overall survival and progression-free survival rates. Because of their low expression of somatostatin receptors, poorly differentiated PanNECs are not highly responsive to peptide receptor radionuclide therapy. Patients with advanced PanNECs have poor prognosis after conventional treatments fail. Targeted therapy and immunotherapy combined with chemotherapy are worthy of further exploration for patients with high-grade PanNECs.

## Multiomics sequencing research on PanNETs

PanNETs are divided into NET-G1 (low grade), NET-G2 (intermediate grade), and NET-G3 (high grade), according to their Ki67 proliferative indexes and mitotic counts. Through comparison of changes in molecules in different grade tumors, variable molecular types in tumors have been defined, thus suggesting that certain important molecules may drive tumor development and partially account for PanNET genetics and pathogenesis. Although the molecular mechanisms underlying PanNETs remain unclear, multiomics sequencing can indicate differences at the DNA, RNA, and protein levels, *via* genomics, transcriptomics, metabonomics, and other omics methods. Thus, application of such sequencing methods may enable the mapping of PanNETs in the multiomics landscape.

### Genomics

Genomics research is key to elucidating the molecular mechanisms underlying tumorigenesis and development, and it is a prerequisite for personalized medicine. In recent years, the rapid development of multiomics sequencing has enhanced understanding of PanNETs at the molecular level. Previous studies have shown that the most frequently mutated gene in PanNETs is MEN1, followed by death domain associated protein (DAXX) and alpha-thalassemia-X-linked intellectual disability syndrome (ATRX)^4^. Notably, DAXX and ATRX mutations are mutually exclusive, and therefore do not occur simultaneously in the same tumor. In addition, 61% of patients with PanNETs have abnormal telomeres. Those cases have either ATRX or DAXX gene mutations or their protein losses, thus indicating that ATRX and DAXX proteins may be involved in chromatin remodeling of telomeres^5^.

Tumor mutational burden (TMB) is a novel bio-maker in tumors. Patients with high TMB show more favorable outcomes after receiving immune checkpoint inhibitor therapy than patients with low TMB. Whole genome sequencing of 102 PanNET specimens has revealed the mutational characteristics, among which the most notable mutation was G:C>T:A inversion in the base excision repair gene MUTYH, accompanied by loss of heterozygosity. PanNETs have a lower mutational burden than pancreatic ductal adenocarcinoma and thus are more likely to less respond to immune checkpoint inhibitor treatment^6^.

No mutations in MEN1, DAXX, or ATRX have been found in insulinomas. However, a study using whole exome sequencing of insulinoma specimens has indicated that 30% of insulinomas had a T372R mutation of Yin Yang 1 (YY1) thus leading decreased expression of YY1, which may be a therapeutic target for insulinomas^7^. Because research on the biological functions of these mutations is lacking, their clinical translational value and applications must be further explored.

### Transcriptomics

Transcriptomic sequencing is used to screen differentially expressed mRNAs and associated signaling pathways in tumors. Genome-wide mRNA gene expression analysis of 32 NF-PanNET samples has revealed upregulation of platelet-derived growth factor receptor beta (PDGFRB), cyclin dependent kinase 4 (CDK4), and cyclin dependent kinase 6 (CDK6) in NF-PanNETs, thus indicating potential subtype-specific antitumor targets^8^. High expression of PDGFRB at both the RNA and protein levels has been reported in PanNETs. PDGFRB regulates hydrostatic pressure, angiogenesis, and uptake of drugs in tumors. Tuberous sclerosis complex (TSC2) and phosphatase and tensin homolog (PTEN), two key inhibitors of the PI3K/Akt/mTOR pathway, have been found to be downregulated in primary tumors *via* quantitative real-time polymerase chain reaction and tissue microarray analysis of PanNETs. These findings further confirm that activation of the mammalian target of rapamycin (mTOR) pathway plays important roles in the progression of tumor growth and invasion^1^. The clinical application of RNAseq or differentially expressed mRNA screening can improve the tumor-targeting specificity of PanNETs, thus increasing treatment efficacy and minimizing off-target toxicity.

### Metabonomics

Tumor metabonomics is a major topic in current sequencing studies. Typical cancer metabolism refers to a variety of metabolic reprogramming events during tumor formation and progression, including glycolysis, fatty acid metabolism, amino acid metabolism, oxidative phosphorylation, and redox metabolism^9^. Increased uptake of glucose has been associated with higher grade PanNETs, thus suggesting the important role of glycolysis in advanced PanNETs. A recent genome-wide CRISPR–Cas9-based knockout screen has indicated that dihydroorotate dehydrogenase (DHODH), the rate-limiting enzyme in de novo pyrimidine synthesis, is a new target protein of MEN1^10^. Loss of MEN1 increases the levels of pyrimidine metabolites by promoting the transcription, and upregulating the expression, of DHODH. Further clinical trials have indicated that leflunomide, a potent DHODH inhibitor, has favorable efficacy as a first-line treatment for MEN1 deficiency-associated PanNETs. Our recent study has indicated that MEN1 inhibits the activation of mTOR, a central hub of metabolism, and increases monounsaturated fatty acid metabolism *via* suppression of stearoyl-CoA desaturase 1 (SCD1) in PanNETs^11^. These results suggest that MEN1 also exerts an important function in PanNET metabolic remodeling, and that targeting MEN1 deficiency-driven metabolic pathways may be a promising treatment strategy for patients with PanNETs.

### Somatic copy number variation

Somatic copy number variation refers to the rearrangement of the genome, and increases or decreases in the copy numbers of large segments of the genome with a length exceeding 1 kb. These variations typically comprise deletion and duplication at the submicroscopic level. Copy number variation is ubiquitous in human normal tissues, accumulates with age, and is considered a precancerous phenomenon. For tumors, copy number variation may occur in a tissue-specific context. For example, chromosomal aberrations are more common in well-differentiated PanNETs than in other tumors. In particular, an elevated chromosome 7 copy number is found in 68% of PanNETs, whereas chromosome 21 deletions are rare. A recent study has reported the mutational landscape and the copy number variation patterns from sequencing of 84 insulinoma and 127 NF-PanNETs^12^. NF-PanNETs were divided into 3 forms according to copy number variation, and patients with variable copy number were found to have high risk of tumor recurrence. Thus, somatic copy number variation may be a useful biomarker for predicting diagnosis and prognosis in PanNETs, and may reflect tumor genomic integrity and vulnerability to anticancer therapy.

### Other omics

Beyond genomics and transcriptomics, progress in other omics research in PanNETs has been relatively slow, probably because of the rarity of specimens. Overall proteomics of PanNETs has not been reported. In recent years, integrated multiomics analysis based on large clinical specimens has been applied to reveal molecular characteristics and potential therapeutic strategies for multiple tumors. Because PanNETs have high heterogeneity in genomic mutations and the tumor microenvironment, given their high aggressiveness and poor prognosis, most previous studies have focused on detecting the tumor genome and transcriptome, and performing corresponding stratification according to tumor molecular type. However, the exploration of molecular type and therapeutic targets according to these studies has been insufficient to achieve optimal clinical benefits for patients, because of individual differences, whereas an integrated multiomics strategy would more accurately characterize patients at the multi-dimensional molecular level and better identify new molecular subtypes of PanNETs. Thus, future integrated multiomics sequencing can enable comprehensive systematic understanding of disease development mechanisms, heterogeneity, prognosis monitoring, and personalized treatment strategies for PanNETs.

## Research on the PanNET tumor microenvironment

Tumor microenvironment research is another hotspot in PanNET research. The occurrence and development of tumors result from interactions between genetic mutations and the surrounding environment. In-depth study of the tumor microenvironment can aid in understanding the biological characteristics of PanNETs.

### Immune microenvironment

As immune background research advances, the infiltration of immune cells, such as B cells, T cells, natural killer cells, dendritic cells, mast cells, and macrophages, has been observed in PanNET tissues and associated with clinical characteristics. For example, few T cells are found in liver metastatic lesions, thus indicating that the cytotoxic immune microenvironment might differ between primary and metastatic tumors. Low mast cell density has been correlated with higher grades and advanced stages of pancreatic neuroendocrine neoplasms^13^. Patients with pancreatic neuroendocrine neoplasms, with a high density of mast cells and low CD3+ T cells, have been found to have prolonged progression-free survival. The addition of macrophages to the PanNET microenvironment has been found to increase the expression of cytokines and chemokines, such as IL-1β, IL-6, monocyte chemoattractant protein-1 (MCP-1), and tumor necrosis factor alpha (TNF-α) in tumor cells, and to promote tumor progression and immunosuppression in the tumor microenvironment^14^.

Tertiary lymphatic structures are ectopic lymphoid organs that develop in nonlymphoid tissues at sites of chronic inflammation. As special components of the tumor immune microenvironment, tertiary lymphoid structures play important roles in recruiting lymphocytes and are associated with good clinical outcomes in a variety of tumors. Our institutional cohort study of NF-PanNETs has indicated that the presence of tertiary lymphatic structures is an independent protective factor in the prognosis of resectable G1/G2 NF-PanNETs^15^, but the biological importance of these tertiary lymphoid structures in PanNETs remains unclear. More importantly, a recent study has used a Gene Expression Omnibus dataset of 158 PanNET cases to summarize immune infiltration and identify immune-associated features *via* the least absolute shrinkage and selection operator regression model. That study constructed the first NET immunoscore system for Pan-NET (ISpnet) by using statistically significant survival predictors from the training cohort (125 cases)^16^. Line maps based on ISpnet and independent clinical risk factors may help monitor the risk of tumor early recurrence, identify high-risk patients requiring adjuvant therapy, and provide an ancillary guide for patients with PanNETs who may benefit from clinical immunotherapy.

The immune checkpoint receptor programmed death protein 1 (PD-1) and its ligands programmed cell death ligand 1 (PD-L1)/programmed cell death ligand 2 (PD-L2) play key roles in the formation of the immunosuppressive microenvironment. High heterogeneity in PD-L1 expression has been reported in various neuroendocrine tumors^6^; for example, PD-L1 is expressed in 7.4% of PanNETs but almost no small intestine neuroendocrine tumors. Because high expression of PD-1/PD-L1 is associated with a strong response to immunotherapy, including anti-PD-1/PD-L1 drugs, PD-1/PD-L1 expression might serve as both a prognostic factor and a predictor of immunotherapy efficacy in PanNETs.

### Tumor-associated fibroblasts

Fibroblasts, a component of the tumor microenvironment, play important roles in the malignant progression of cancer by secreting growth factors and chemokines, producing extracellular matrix, and promoting the recruitment of endothelial cells. They transform into activated cancer-associated fibroblasts (CAFs) during tumorigenesis. Little research has examined CAFs in PanNETs. However, as early as the 1990s, conditioned medium from BON-1 cell lines was found to stimulate fibroblast colony growth^17^. Further studies have indicated that BON-1-induced fibroblast proliferation is associated with transforming growth factor-beta (TGF-β) release, and fibroblast-mediated IL-6 and VEGF secretion is significantly increased by TGF-β. In addition, multiple studies have shown that TGF-β and its receptors are coexpressed in tumor and stromal cells of NETs, and that TGF-β1, TGF-β2, and TGF-β3 may drive fibroblast synthesis of α-smooth muscle actin^18^. Because abundant CAFs around PanNETs contribute to chemotherapy tolerance, and the interaction between tumor cells and CAFs further promotes tumor development and progression, new CAF-targeted therapies and rational combination approaches must be explored in the future.

### Tumor microvascularity

Although PanNETs exhibit high heterogeneity, tumor angiogenesis is a common feature of the PanNET microenvironment, because endocrine gland tissue has many microvessels composed of porous endothelial cells that promote hormone secretion and release into the bloodstream. Vascular formation is a multistep process modulated by the balance of multiple proangiogenic and antiangiogenic factors. Vascular endothelial growth factor (VEGF) and vascular endothelial growth factor receptor (VEGFR) are often overexpressed in PanNETs compared with normal tissues. VEGF mediates angiogenesis by binding 2 highly related tyrosine kinase receptors, VEGFR-1 and VEGFR-2, which are expressed primarily by endothelial cells and promote angiogenesis after activation. Notably, plasma circulating VEGF concentrations are higher in patients with PanNETs with metastatic rather than nonmetastatic tumors. However, this finding does not necessarily indicate that VEGF is sufficient for tumor cell metastasis, because angiogenic markers are expressed highly in well-differentiated and low-grade tumors compared with high-grade and/or undifferentiated tumors, in a scenario known as the neuroendocrine paradox^19^. The molecular mechanism of PanNET microangiogenesis is relatively clear, and VEGF pathway inhibitors, such as bevacizumab, sunitinib, and sulfatinib, have been found to elicit clinical effects. However, VEGF pathway inhibitors confer only a temporary beneficial effect at best, and tumor growth and progression recur soon after drug withdrawal.

## Genetics and pathogenesis of PanNETs and future perspectives

In recent years, the incidence of PanNETs has increased each year, and these malignant tumors threaten human life and health. With advances in next-generation sequencing, particularly the application of multiomics sequencing, such as whole-exome sequencing and high-throughput transcriptomics sequencing, the genetic map of PanNETs has been determined, yet remains insufficient to explain tumor heterogeneity and the underlying molecular evolution trajectory^20^. The genetics and pathogenesis of PanNETs remain unclear. The application of new sequencing technologies, e.g., single-cell sequencing and spatial multiomics sequencing, in PanNETs could provide new means of understanding the molecular mechanisms of PanNETs. Single-cell sequencing technology can be used to define the functions of different cell types in tumors or to elucidate the tumor microenvironment. Spatial multiomics sequencing can be applied in the exploration of tumor spatial and temporal changes at the molecular level, and is expected to advance in-depth understanding of the high heterogeneity of PanNETs and to guide individualized treatment for patients.

**Figure 2:**
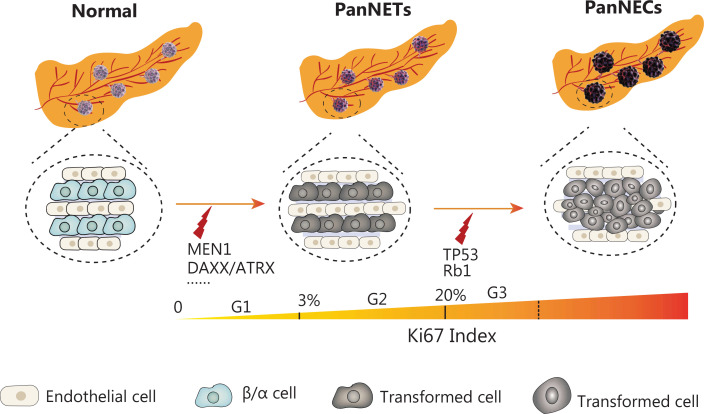
Proposed mechanisms underlying the pathogenesis of pancreatic neuroendocrine tumors. PanNETs arise from mutations in MEN1, DAXX, ATRX, and other pathway genes e.g., mTOR and PTEN. Mutations in TP53 (p53 suppressor gene) and Rb1 (retinoblastoma tumor suppressor gene) are seen in advanced PanNETs, and may drive the pathological advancement of tumors.

On the basis of current sequencing research, we speculate that the occurrence and development of PanNETs involve a multistage evolutionary process. First, mutations in tumor suppressor genes, including MEN1, DAXX, and ATRX, lead to the initiation of PanNETs; subsequently, p53 inactivation mutations occur over time, thus gradually increasing the pathological grade of PanNETs (G1→G2, G2→G3) and further promoting the differentiation of PanNETs into high grade neuroendocrine carcinomas, thereby promoting the malignant advancement of tumors (**Figure 2**). We expect future research to integrate new sequencing technologies and combine them with tumor malignant biology behaviors to reveal the pathogenesis of PanNETs, search for new therapeutic targets, and improve patient prognosis according to the molecular evolutionary trajectory of PanNETs.
